# [Corrigendum] Cell density-dependent regulation of p73 in breast cancer cells

**DOI:** 10.3892/ijo.2026.5898

**Published:** 2026-05-25

**Authors:** Chaitali Tophkhane, Shihe Yang, Zhizhuang Joe Zhao, Xiaohe Yang

Int J Oncol 35: 1429-1434, 2009; DOI: 10.3892/ijo_00000461

Subsequently to the publication of the above article, an interested reader drew to the authors' attention that the control β-actin blots shown for the western blots portrayed in Figs. 1B and 2B were strikingly similar after a slight adjustment was made to one set of the bands, even though the experiments shown for the p73β protein were different in these figure parts.

After re-examining their original data, the authors have realized that the β-actin blots correctly shown for Fig. 2B had inadvertently been included in Fig. 1B. The revised version of Fig. 1, now showing the β-actin blots that were correctly associated with Fig. 1B, is shown opposite. The authors are grateful to the Editor of *International Journal of Oncology* for allowing them this opportunity to publish a Corrigendum, and all the authors agree to its publication. Note that this error did not grossly affect either the results or the conclusions reported in this study; furthermore, the authors apologize to the readership for any inconvenience caused.

## Figures and Tables

**Figure 1:**
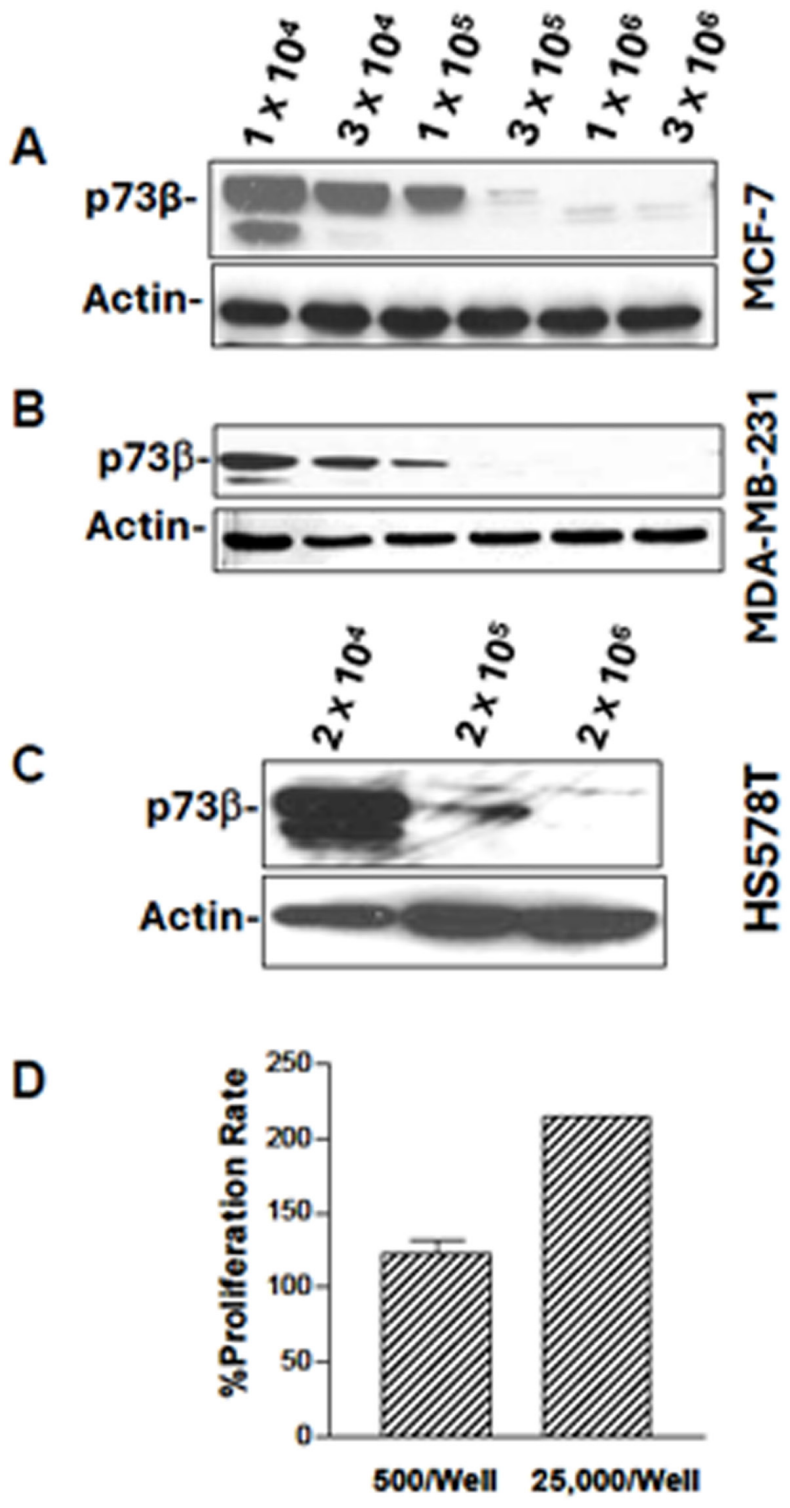
Cell density regulates p73 expression in breast cancer cells (A-C). MCF-7 (A), MDA-MB-231 (B) and HS578T (C) cells were seeded at different densities and cultured for 48 h. Protein levels of p73β were analyzed with Western blot (the p73 antibody was AB3). (D) Proliferation rate of MCF-7 cells at different densities. The cells were inoculated in a 24-well plate at 500/well or 25,000/well. Cell proliferation rate was calculated based on SRB absorbance of 48 h over 24 h. ^*^p<0.01.

